# Wortmannin Reduces Insulin Signaling and Death in Seizure-Prone *Pcmt1^−/−^* Mice

**DOI:** 10.1371/journal.pone.0046719

**Published:** 2012-10-05

**Authors:** Kennen B. MacKay, Jonathan D. Lowenson, Steven G. Clarke

**Affiliations:** Department of Chemistry and Biochemistry and the Molecular Biology Institute, University of California Los Angeles, Los Angeles, California, United States of America; National Cancer Institute, United States of America

## Abstract

L-isoaspartyl (D-aspartyl) *O*-methyltransferase deficient mice (*Pcmt1^−/−^*) accumulate isomerized aspartyl residues in intracellular proteins until their death due to seizures at approximately 45 days. Previous studies have shown that these mice have constitutively activated insulin signaling in their brains, and that these brains are 20–30% larger than those from age-matched wild-type animals. To determine whether insulin pathway activation and brain enlargement is responsible for the fatal seizures, we administered wortmannin, an inhibitor of the phosphoinositide 3-kinase that catalyzes an early step in the insulin pathway. Oral wortmannin reduced the average brain size in the Pcmt1^−/−^ animals to within 6% of the wild-type DMSO administered controls, and nearly doubled the lifespan of Pcmt1^−/−^ at 60% survival of the original population. Immunoblotting revealed significant decreases in phosphorylation of Akt, PDK1, and mTOR in Pcmt1^−/−^ mice and Akt and PDK1 in wild-type animals upon treatment with wortmannin. These data suggest activation of the insulin pathway and its resulting brain enlargement contributes to the early death of Pcmt1−/− mice, but is not solely responsible for the early death observed in these animals.

## Introduction

Successful aging is dependent upon an organism’s ability to protect its macromolecular machinery over time, and if this is not sufficient, to repair or replace that machinery [1], [2], [3]. Protein damage due to the spontaneous deamidation and isomerization of asparagine and aspartic acid residues, respectively, can build up over time and lead to alterations in tertiary protein structure and enzyme activity [4]. Additionally, isoaspartyl formation can act as an age-timed molecular switch altering enzyme function [5]. Organisms respond to such damage with the L-isoaspartyl (D-aspartyl) *O*-methyltransferase (PCMT1), a protein repair methyltransferase that initiates the conversion of L-isoaspartyl residues to normal L-aspartyl residues [4]. Pcmt1 is conserved from bacteria to humans and overexpression of this protein has been linked to extended lifespan in *Escherichia coli*, *Caenorhabditis elegans,* and *Drosophilia melanogaster* [6], [7], [8].

Although there are no reports of Pcmt1 overexpression in mammals, genetic deletion of this enzyme in mice leads to a significant increase in isoaspartyl residues in intracellular proteins [9], [10]. Additionally these mice display reduced overall body size, enlarged brains, and have been reported to die at approximately 45 days of age from tonic-clonic seizures [11], [12], [13]. Although the cause of the seizure and reduced body size phenotypes remains to be resolved, the enlarged brain size is thought to be attributed, at least in part, to aberrantly increased insulin signaling in neuronal tissues [12], [13], [14]. This theory is reinforced by data showing similar effects in mice genetically modified to have increased insulin signaling [15]. Down-regulation of PCMT1 in human epileptic hippocampus suggests there may be a conserved role of PCMT1 in seizure disorders [16].

A conserved link between the insulin signaling pathway and the isoaspartyl repair methyltransferase also appears in the nematode *C. elegans*, where lifespan extension due to overexpression of the methyltransferase requires the activity of the DAF-16 transcription factor that is inactivated by insulin signaling [7], [17]. On the other hand, repair methyltransferase-deficient mutants of *C. elegans* demonstrate diminished expression of at least some DAF-16 target genes [7]. Consistent with these observations, the loss of the repair methyltransferase in *C. elegans* results in a reduced starvation response and decreased lifespan under stress [18]. It has been hypothesized that either the accumulation of damaged proteins in methyltransferase knockouts acts as a direct switch activating insulin signaling or that the methyltransferase may directly interfere with the insulin-signaling pathway independent of isoaspartyl accumulation [7].

The sudden death phenotype of *Pcmt1^−/−^* mice clearly precludes their use as an aging model and prevents the discovery of the role of isoaspartyl accumulation in aging. Some progress has been made in developing *Pcmt1^−/−^* mice expressing transgenic L-isoaspartyl methyltransferase on a neuron-specific promoter [19]. These mice express low levels of this enzyme in the brain and display increased survival. In these mice, there appears to be a proteolytic system that engages at approximately 100 days of age to compensate for the rising level of L-isoaspartyl-containing proteins [19]. In *C. elegans* this link is further reinforced by evidence suggesting that the absence of the repair methyltransferase reduces autophagy, indicating a direct link between PCMT1 and protein turnover [20].

We hypothesized that the aberrant growth signaling pathways and/or the enlarged brains in repair methyltransferase-deficient mice could be contributing to the seizure phenotype. Although the underlying cause of the increased brain size in *Pcmt1^−/−^* animals is currently unknown, the aberrant insulin signaling in the brains of these animals is theorized to be the leading cause of the enlarged brain size observed [12]. In this study we sought to knock down the insulin-signaling cascade through the use of the phosphoinositide 3-kinase (PI3K) inhibitor wortmannin [21]–[22]. PI3K is an essential element of the insulin cascade responsible for recruiting the AGC family of kinases, including Akt, PDK1 and mTORC2, to the membrane where Akt is phosphorylated and activated [23] [17]. If the increased activity of the insulin-signaling pathway is indeed involved in the seizure phenotype, inhibition of PI3K may reduce the brain size of *Pcmt1^−/−^* animals, limit seizure activity, and prolong their lifespan. We directly tested this hypothesis by maintaining mice on wortmannin and tracking their growth, lifespan, and insulin-signaling activation. Our results suggest the isoaspartyl methyltransferase may affect insulin signaling at or after the PI3K-dependent activation of Akt. We show that reduction of PI3K activity in *Pcmt1^−/−^* mice prevents the insulin-signaling cascade from exerting its downstream pleiotropic effects and establishes the aberrantly increased insulin signaling in the brains of these animals as the causative factor for their increased brain size. Additionally, wortmannin partially ameliorated seizure onset and extended lifespan in Pcmt1^−/−^ animals.

## Methods

### Ethics Statement

This study was performed in accordance with animal use protocols approved by the UCLA Animal Research Committee (Protocol 1993-109-62). Mice were scheduled to be euthanized if they met any early removal criteria (kyphosis, lack of grooming behavior). However, this did not occur with any of the animals in our study.

### Animal Husbandry

Mice were kept on a 12-hour light/dark cycle and allowed *ad libitum* access to water and NIH-31 7013 pellet chow (18% protein, 6% fat, 5% fiber, Harlan Teklad, Madison, WI). *Pcmt1^−/−^* animals were generated through breeding of *Pcmt1^+/−^* animals as reported previously [9], [12]. These animals have been interbred for fifteen years to obtain a genetically homogeneous population. *Pcmt1^−/−^* and *Pcmt1^+/+^* offspring were used in this study. Experimental animals were weaned at 21 or 22 days of age; and we then began the administration of wortmannin or control solutions once per day until the mice reached 44 days of age. At this time, they were fasted for 15 hours and sacrificed by carbon dioxide asphyxiation for tissue extraction. Wortmannin (Alexis Biochemicals, San Diego, CA; lot 24089) was stored at −20°C in a 25 mg/ml solution in DMSO. Immediately prior to administration, mice were weighed. A fresh aliquot of wortmannin was diluted 1∶10 in a grape-flavored sugar-based drink (Inter-American Products, Cincinnati, OH) and animals were administered oral doses using a calibrated Gilson P20 Pipetman containing 1.5 mg drug/kg body weight in the evening hours. Control animals were given a corresponding 1∶10 dilution of DMSO in grape drink at the same time. A fresh pipet tip was used for each animal, and the mouse was held until the solution was observed to be swallowed. Animals were administered either drug or DMSO in a blinded fashion based on cage numbers and animal markings without knowledge of the genotype. Animals were housed in same-sex cages with two or three other mice.

### Brain Extraction and Western Blotting

Following their final dose of wortmannin or control solution, 44 day-old mice were fasted overnight for 15 h and subsequently euthanized on their 45^th^ day of age in a CO_2_ chamber prior to surgical brain removal. Brain tissue (excluding the olfactory bulbs) was dissected, weighed, and added to 3 ml/g of RIPA buffer (50 mM Tris-HCl pH 8, 150 mM NaCl, 0.5% sodium deoxycholate, 1% Triton X-100, 1 mM PMSF) with phosphatase (HALT, Thermo-Pierce, Rockford, IL) and protease inhibitors (Complete, Roche, Mannheim, Germany) and homogenized using a Polytron homogenizer with a PTA-7 generator. The protein concentration of the crude extracts was determined after trichloroacetic acid precipitation by the Lowry method [24]. Aliquots containing 20 µg of protein were added to 10 µl of a 2X SDS-sample loading buffer (100 mM Tris-HCl, pH 6.8, 200 mM β-mercaptoethanol, 4% SDS, 0.1% bromophenol blue, 20% glycerol) and then brought to a final volume of 20 µl with water and heated for 5 min at 100°C. The samples were then loaded into lanes of twelve-well, 10 cm by 10 cm, 4–12% RunBlue SDS gels (Expedeon, San Diego, CA) in an Invitrogen XCell SureLock Mini-Cell apparatus along with parallel lanes of rainbow molecular weight markers (RPN-800V, GE Healthcare, Buckinghamshire, England). Electrophoresis was performed at 180 V for 1 h. Proteins were transferred from gels to PVDF membranes (Amersham Hybond-P, GE Healthcare) by electrophoretic transfer at 25 V for 3 h using the Invitrogen Blot Module and NuPAGE transfer buffer (Invitrogen, Grand Island, NY). Membranes were blocked overnight using 5% bovine serum albumin and then probed with the primary antibodies diluted in TBS-T buffer as described in Table 1. After the blot was washed in TBS-T buffer, it was incubated with horseradish peroxidase-labeled secondary antibodies as described in Table 1. Peroxidase activity was visualized after treating the blot with ECL Prime Chemiluminescent Agent (GE Healthcare) and detected on Hyblot CL film (Denville, Metuchen, NJ). Exposure times were optimized to allow linear responses. Film densitometry was performed using ImageJ densitometry software.

**Table 1:** Source of Antibodies and Immunoblotting Protocols.

Target	Name	Source	Dilution	Incubation time	Temperature	Polypeptide size
p-S241-PDK1	Phospho-PDK1 (Ser241) (C49H2) Rabbit mAb #3438	Cell Signaling	1∶10,000	1 h	25°C	58 kDa
Akt (pan)	Akt (pan) (C67E7) Rabbit mAb #4691	Cell Signaling	1∶10,000	1 h	25°C	60 kDa
p-S473-Akt	Phospho-Akt (Ser473) (D9E) XP Rabbit mAb #4060	Cell Signaling	1∶15,000	1 h	25°C	60 kDa
p-T308-Akt	Phospho-Akt (Thr308) (C31E5E) Rabbit mAb #2965	Cell Signaling	1∶10,000	1 h	25°C	60 kDa
p-S2481-mTOR	Phospho-mTOR (Ser2481) Antibody #2974	Cell Signaling	1∶10,000	18 h	25°C	289 kDa
p-S2448-mTOR	Phospho-mTOR (Ser2448) (D9C2) XP Rabbit mAb #5536	Cell Signaling	1∶10,000	18 h	25°C	289 kDa
mTOR	mTOR (7C10) Rabbit mAb #2983	Cell Signaling	1∶10,000	24 h	25°C	289 kDa
GAPDH	GAPDH (14C10) Rabbit mAb	Cell Signaling	1∶40,000	1 h	25°C	37 kDa
PCMT1	Anti-PCMT1 cultured in rabbit (non-commercial)	Gift from Dr. Mark Mamula	1∶1000	1 h	4°C	25 kDa
Anti-Rabbit	Anti-Rb Goat HRP conjugated secondary (ab6721)	Abcam	1∶100,000	1 h	25°C	NA

### Quantitation of L-isoaspartyl Residues in Soluble Mouse Brain Extracts

The content of L-isoaspartyl residues in soluble mouse brain proteins was determined with an assay similar to that used previously [19]. Briefly, recombinant human L-isoaspartyl methyltransferase was used as a reagent to catalyze the transfer of ^14^C-methyl groups from *S*-adenosyl-[*methyl*-^14^C] methionine to L-isoaspartyl residues. After hydrolysis of the methyl esters formed, ^14^C-methanol was quantified using a vapor diffusion assay. Samples were prepared by diluting the mouse brain crude homogenates described above two-fold with RIPA buffer, centrifugation at 20,800×g for 20 min at 4°C, and collection of the supernatant. The isoaspartyl methyltransferase assay mixture consisted of 5 µL of RIPA buffer containing 2 to 4 µg of protein from the supernatant fraction of *Pcmt1* ^−/−^ brain extract or 20 µg of protein from *Pcmt1^+/+^* brain extract, 10 µM *S*-adenosyl [*methyl*-^14^C]methionine (48.8 mCi/mmol; Amersham Biosciences), 2.24 µg of recombinant human L-isoaspartyl methyltransferase (8944 pmol/min/mg protein), 160 mM bis-Tris-HCl buffer at pH 6.4 in a final volume of 40 µl. After a 3 h incubation at 37°C, ^14^C-methyl ester content was quantitated as described [19]. All samples were assayed in triplicate. As a negative control, the brain sample was substituted with an equal volume of RIPA buffer. Radioactivity measured here was subtracted from the protein-containing samples. As a positive control, the brain sample was replaced with RIPA buffer and L-isoaspartyl-containing ovalbumin (80 mg/mL; Sigma-Aldrich A5503) dissolved in the bis-Tris buffer [19]. The positive control demonstrated that the RIPA buffer in the assay did not inhibit the recombinant isoaspartyl methyltransferase, and that there was enough methyltransferase activity and *S*-adenosyl[*methyl*-^14^C]methionine in each incubation to methylate more than 25-fold more L-isoaspartyl residues than were detected in the mouse brain samples.

## Results

### Decreased Body Weights in Wortmannin-treated Mice

**Figure 1:**
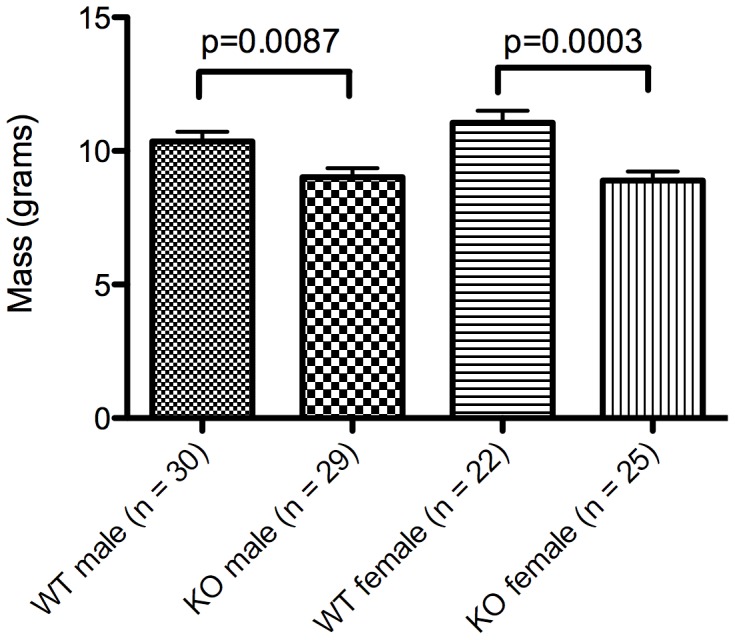
Body weight of wild-type and *Pcmt1^−/−^* mice at time of weaning. *Pcmt1^−/−^* mice are significantly smaller than their wild-type littermates when they are weaned at 21 or 22 days of age. The average weight +/− the standard deviation is shown as well as p-values calculated by Student’s t-test.

**Figure 2:**
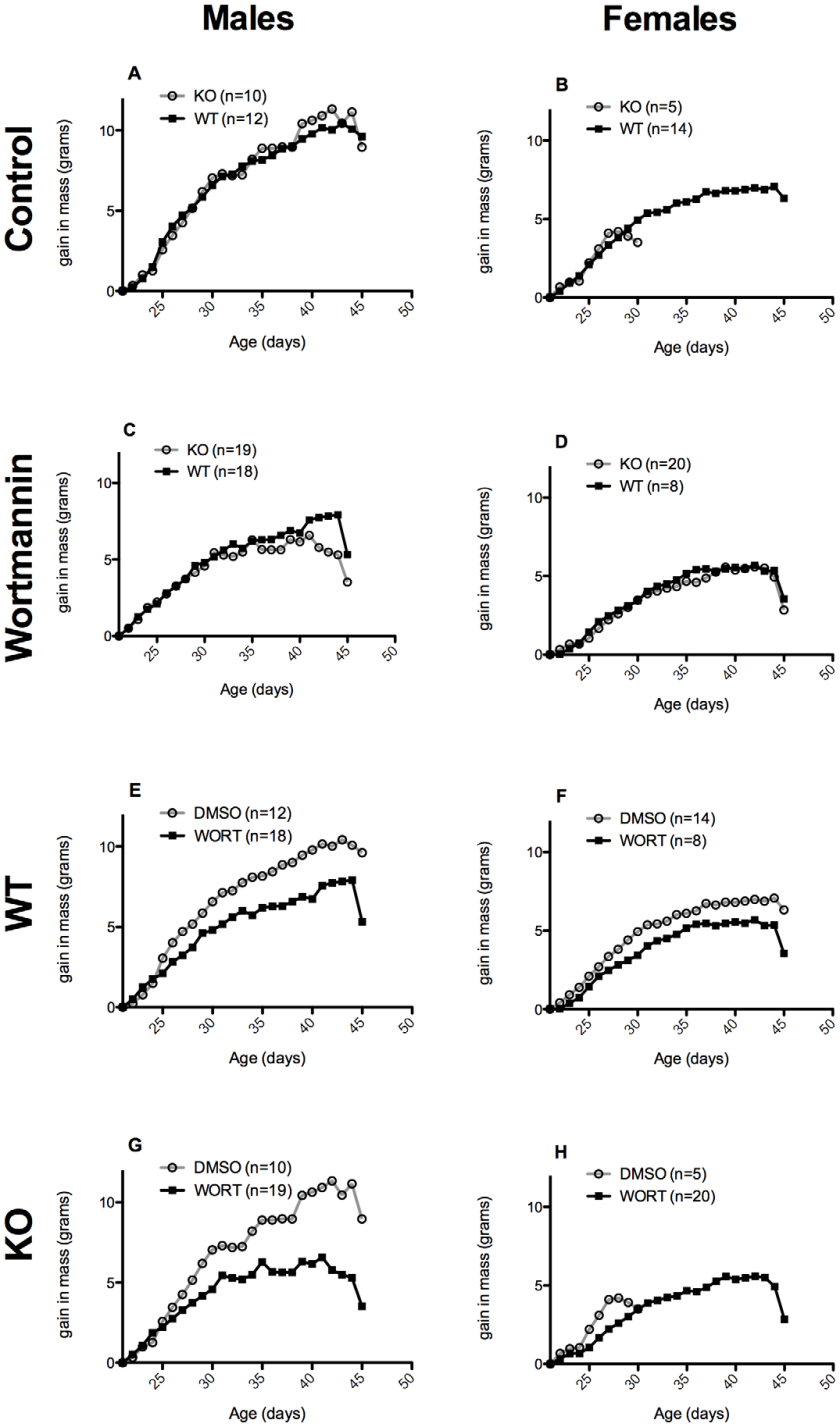
Effect of wortmannin (WORT) on the post-weaning weight gain of wild-type (WT) and protein L-isoaspartyl methyltransferase-deficient (*Pcmt1^−/−^*) (KO) mice. In panels A-D, wild-type weight gains are shown in closed squares and *Pcmt1^−/−^* weight gains are shown in open circles. In panels E-H, weight gains of wortmannin-treated animals are shown in closed squares while those of DMSO-treated control mice are shown in open circles. In all cases animals treated with wortmannin showed significant growth retardation compared to their sex or genotype matched control counterparts.

In an effort to test whether inhibition of the increased insulin signaling in *Pcmt1^−/−^* mice may alleviate the early death and growth phenotypes displayed by these animals, we treated groups of mice with daily 1.5 mg/kg oral doses of the PI3K inhibitor wortmannin beginning 21 or 22 days after birth at the time of weaning [22], [25]. This dose was chosen based on two published reports that oral wortmannin administration at similar dosage levels significantly reduced β-amyloid deposition in an Alzheimer’s disease model mouse [26] and tumor growth in a mouse cancer model [27], and had no adverse effects on these animals. Just prior to drug treatment, we confirmed the smaller size of *Pcmt1^−/−^* mice compared to their *Pcmt1^+/+^* littermates (Figure 1) as has been previously reported [9]. Although wortmannin has been used orally as an inhibitor of the kinase in mice [26], [27] and in rats [28], [29], it has not been established if such treatment would inhibit insulin-signaling activity. We thus treated *Pcmt1^−/−^* and wild-type mice as described above with wortmannin dissolved in DMSO, or DMSO alone, both diluted ten-fold in a grape flavored sugar drink. This drug administration was not done by gavage; instead, these mice were observed to swallow the 5–12 µL solution that was placed in their mouth via a Gilson P20 Pipetman. We found that the increase in body weight following weaning was identical for *Pcmt1^−/−^* and *Pcmt1^+/+^* mice when grouped by treatment and sex (Figure 2, panels A-D; Figure S1, panels A-D). Additionally, the body weight of the animals in the control group was similar to those of previously studied mice on the same NIH-31 7013 diet in the absence of DMSO and the sugar drink [9], suggesting that the DMSO and the additional sugar in the drink does not significantly alter growth.

The average body weight of all groups of animals treated with wortmannin decreased significantly regardless of *Pcmt1* genotype (Figure 2, panels E-H; Figure S1, panels E-H). At 44 days of age, male wild-type animals weighed on average 22.1 g in the DMSO control group while animals administered wortmannin weighed on average 17.1 g. This decrease in mass was also seen in the male *Pcmt1^−/−^* animals, which had average weights at this time of 20.5 g (DMSO) and 15.2 g (wortmannin). Female wild-type control and wortmannin groups weighed 18.6 g and 15.8 g respectively. Because no female control *Pcmt1^−/−^* animals on DMSO survived the experimental time period, no data on their final body weight was available; however, female *Pcmt1^−/−^* animals on wortmannin had an average mass of 13.8 g at day 44.

At 44 days, all animals were fasted in preparation for tissue analyses. Animals were fasted in order to ensure there were no differences in food consumption and blood sugar levels prior to analysis, as well as to ensure we were assaying baseline insulin signaling levels. Although statistically insignificant, all sex and genotype paired animal groups administered wortmannin lost a larger percentage of their body mass during overnight fasting than their DMSO control counterparts. In male wild-type animals the body mass loss amounted to 13.0% in the DMSO-treated group but 15.1% in the wortmannin-treated group. Similarly, male *Pcmt1^−/−^* mice lost 10.7% and 13.6% loss for control and wortmannin animals respectively. Finally, in female wild-type animals the losses amounted to 11.2% and 12.0% in DMSO and wortmannin animals respectively.

The large reduction in body weight over the course of drug administration suggests that the oral administration of wortmannin does in fact decrease insulin signaling-related growth, presumably by the inhibition of the PI3K. Attenuation of insulin signaling, through genetic knockouts as well as by RNA interference of pathway components (including PI3K variants), has been shown to generally result in decreased body size and stature [30].

### Decreased Brain Weights in Wortmannin-treated Mice

**Figure 3:**
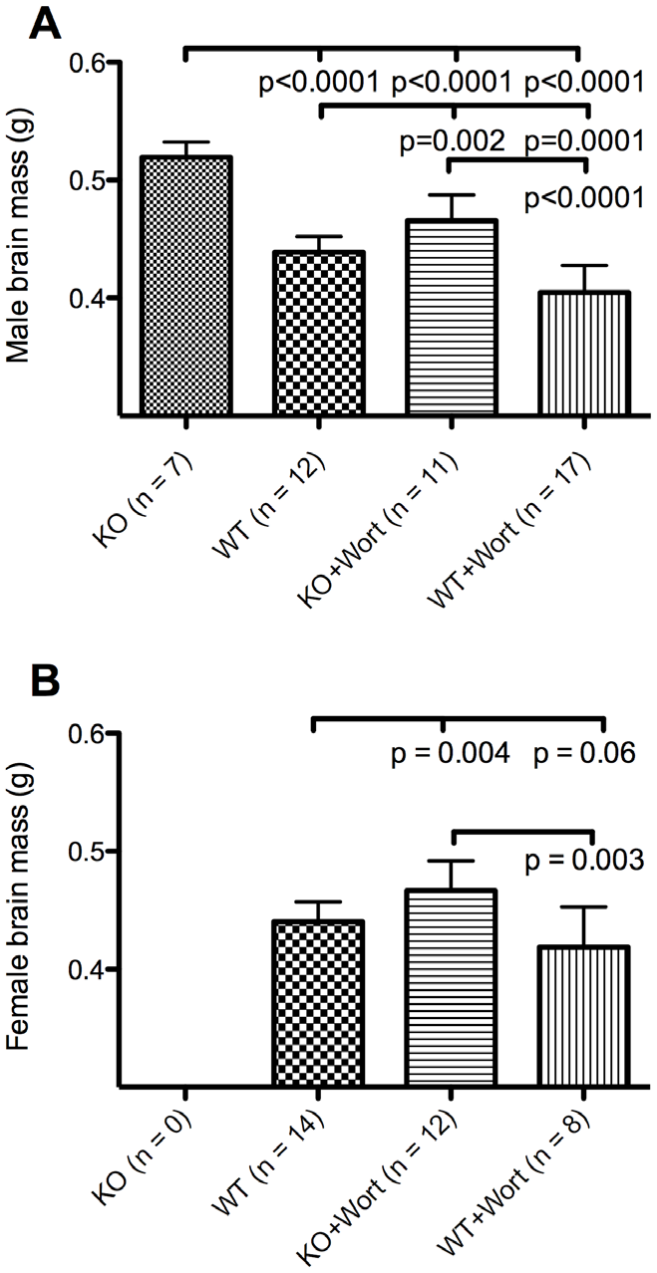
Comparison of brain weights at 45 days of wortmannin (WORT) and control DMSO treated wild-type (WT) and *Pcmt1^−/−^* (KO) mice. Panel A: Male mice. Panel B: Female mice. No female KO mice without WORT survived to 45 days of age. In each case the patterned bars indicate the average of ‘n’ treated animals +/− the standard deviation. The horizontal bars indicate the p values obtained by the Student’s t-test (two-tailed, unpaired) in the indicated comparisons.

Although *Pcmt1^−/−^* animals have a decreased body size they exhibit enlarged brains [10], [12], [14]. Such an increase in brain size was confirmed here in the control group of male mice (lanes 1 and 2 of Figure 3A). In male mice treated with wortmannin, brain size decreased for both *Pcmt1^−/−^* and wild-type animals (Figure 3A, lanes 1 and 3; lanes 2 and 4, respectively). These results confirm the role of insulin signaling in the increased brain size in *Pcmt1^−/−^* animals [12], [13], [14].

Male *Pcmt1^−/−^* animals treated with wortmannin on average had a brain mass 0.06 g less than those in the DMSO control group, while wild-type animals treated with wortmannin had brains on average 0.02 g smaller than control animals. Male *Pcmt1^−/−^* animals thus lost about three times as much brain weight with wortmannin treatment as compared to wild-type animals. Interestingly, male mice of both genotypes treated with wortmannin showed similar losses in body mass: 5 grams for male wild-type animals and 5.3 grams for male *Pcmt1^−/−^* animals. These results suggest that there is an interaction of the insulin-signaling pathway and the protein repair methyltransferase in the brain that may not occur generally in the rest of the body. Female wild-type animals on wortmannin lost on average 0.03 g of brain mass as compared to control treated animals. The lack of female *Pcmt1^−/−^* control animal survivors precludes our ability to make this calculation for female *Pcmt1^−/−^* animals. The tripling in brain weight lost in male *Pcmt1^−/−^* animals upon wortmannin treatment suggests wortmannin is reducing the brain specific insulin signaling in *Pcmt1^−/−^* animals.

Comparing the increase in brain size due to the absence of PCMT1 expression in the DMSO-control *Pcmt1^−/−^* group (0.08 g; lanes 2 and 1 in Figure 3A) with that of the wortmannin-treated group (0.04 g; lanes 4 and 3 in Figure 3A) enables us to map the location of *Pcmt1*’s influence within the insulin-signaling pathway. The fact that wortmannin-treated *Pcmt1^−/−^* animals still display enlarged brains compared to wild-type DMSO-treated animals suggests three possibilities. First, there may be incomplete PI3K inhibition through wortmannin-treatment. Second, the effect of the protein repair methyltransferase may converge on the insulin-signaling pathway downstream of this kinase. Finally, there may be an alternate growth pathway influenced by the repair methyltransferase.

### Lifespan Extension by Wortmannin in *Pcmt1^−/−^* Mice

**Figure 4:**
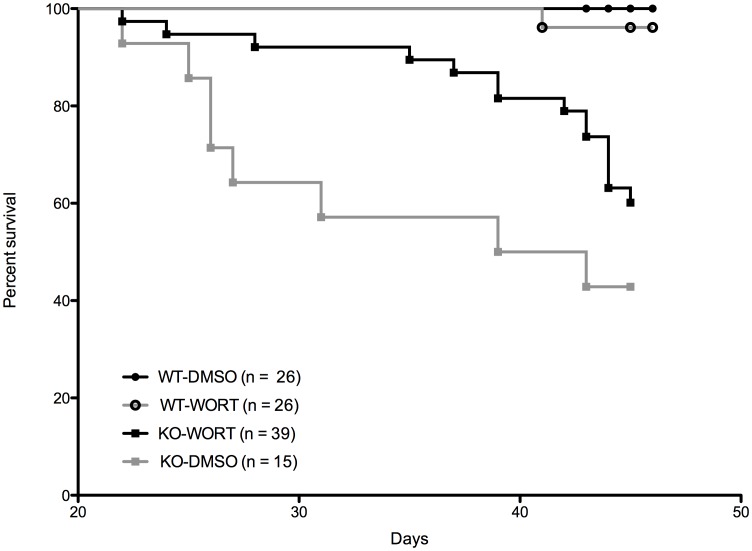
Survival curves of wortmannin (WORT)- and control (DMSO)–treated wild-type (WT) and *Pcmt1^−/−^* (KO) mice. The number of mice (both male and female) in each group is indicated in the legend. The survival of the KO group on wortmannin is significantly longer than the control KO group treated with DMSO according to the Gehan-Breslow-Wilcoxon Test (p = 0.049).

To discover whether wortmannin treatment could alleviate the fatal tonic clonic seizures in *Pcmt1^−/−^* animals, we plotted lifespan data collected over the course of this experiment (Figure 4A). In order to increase sample size and statistical significance, we combined data from male and female animals as data collected during the maintenance of our mouse colony over the last 3 years shows that there is no difference in the survival of male and female *Pcmt1^−/−^* animals (Figure S2). We have now observed that wortmannin-treated *Pcmt1^−/−^* animals live significantly longer than their DMSO-treated control counterparts. As only one wild-type animal died over the experimental period, the effect of wortmannin on wild-type survival remains unknown. The decreased fatalities with wortmannin treatment, presumably due to prolonged time before seizure onset [9], [11], [19], correlates well with the decreased brain size seen in Figure 3.

### Direct Analysis of the Insulin-signaling Pathway in Wortmannin-treated *Pcmt1^−/−^* and *Pcmt1^+/+^* Mice

**Figure 5:**
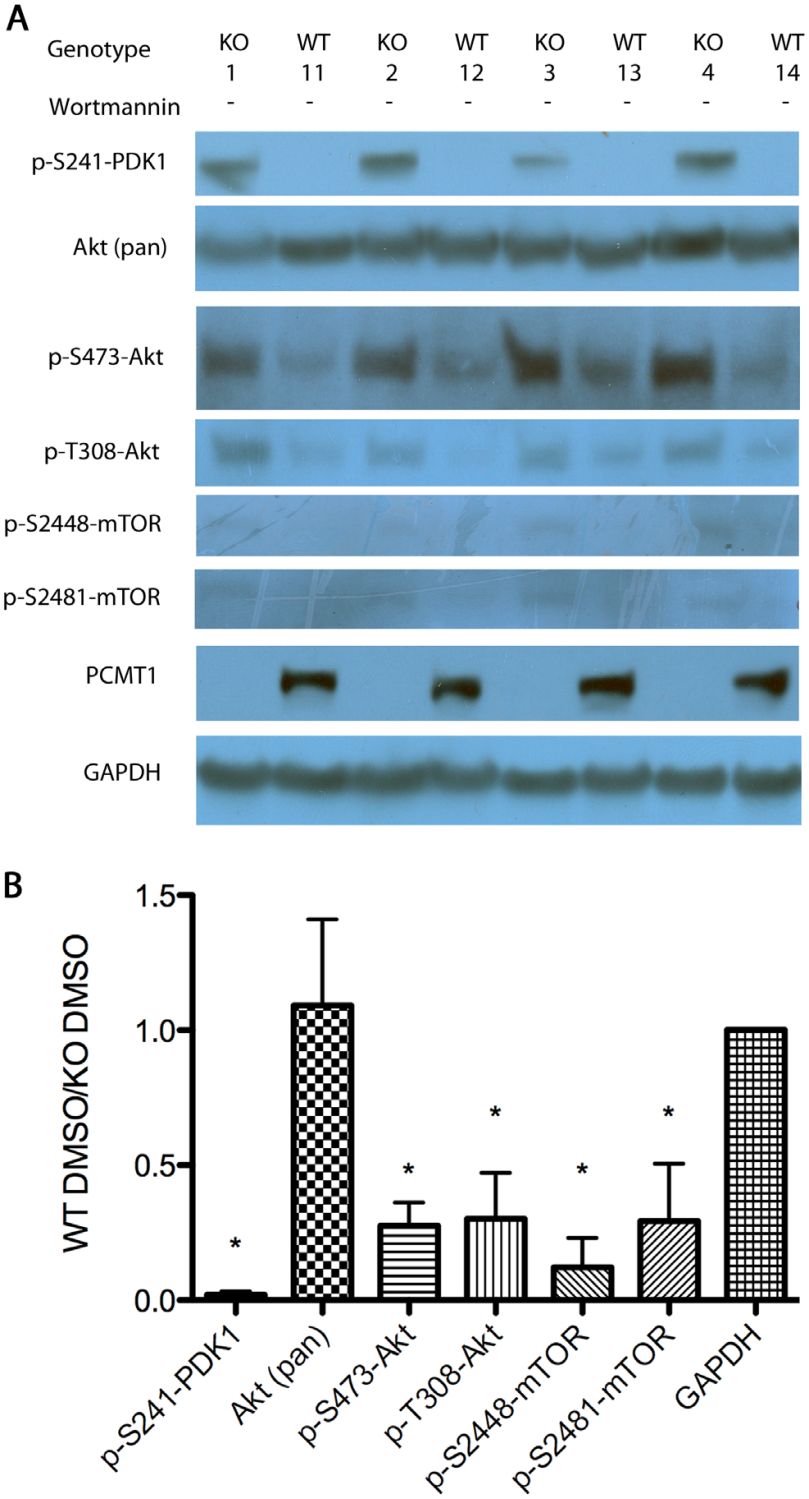
Western blot analysis of the phosphoproteins of the insulin-signaling cascade in homogenized whole-brains from male wild-type (WT) and *Pcmt1^−/−^* (KO) animals in the absence of wortmannin. Panel A: representative Western blots from four KO and four WT animals. Each row represents an independent exposure. Panel B: averaged densitometry results from all animals analyzed (n = 7 for KO; n = 12 for WT), standardized to GAPDH band densities to ensure equal protein loading. The molecular weight of each of the bands as determined in comparison with rainbow markers was consistent with the known weight of the target protein (Table 1). The error bars denote standard deviations; asterisks indicate statistical significance (p<0.05) by Student t-test between the WT and KO samples.

By immunoblotting brain extracts for the activated phosphorylated components of the insulin-signaling system, we first confirmed that insulin signaling was potentiated in DMSO-treated *Pcmt1^−/−^* mice as previously described [12]. Phosphorylation of Ser-241 of PDK1, as well as Ser-473 and Thr-308 of Akt were each seen to be significantly increased in the *Pcmt1^−/−^* mice (Figure 5), suggesting that the DMSO treatment in our current experiments does not significantly alter brain phosphorylation patterns from the untreated animals described previously [12]. No change in total Akt protein levels was observed. Additionally we quantitated phosphorylation sites of Ser-2448 and Ser-2481 of mTOR. The former is associated with the active mTORC1 complex, the latter with the active mTORC2 complex [31]. Both mTOR phosphorylation sites appear to increase significantly in the *Pcmt1^−/−^* extracts, suggesting there is an overall increase in activated mTOR kinase in both mTORC1 and mTORC2 complexes. PCMT1 was also assayed in order to confirm the genotype of the *Pcmt1^−/−^* animals.

**Figure 6:**
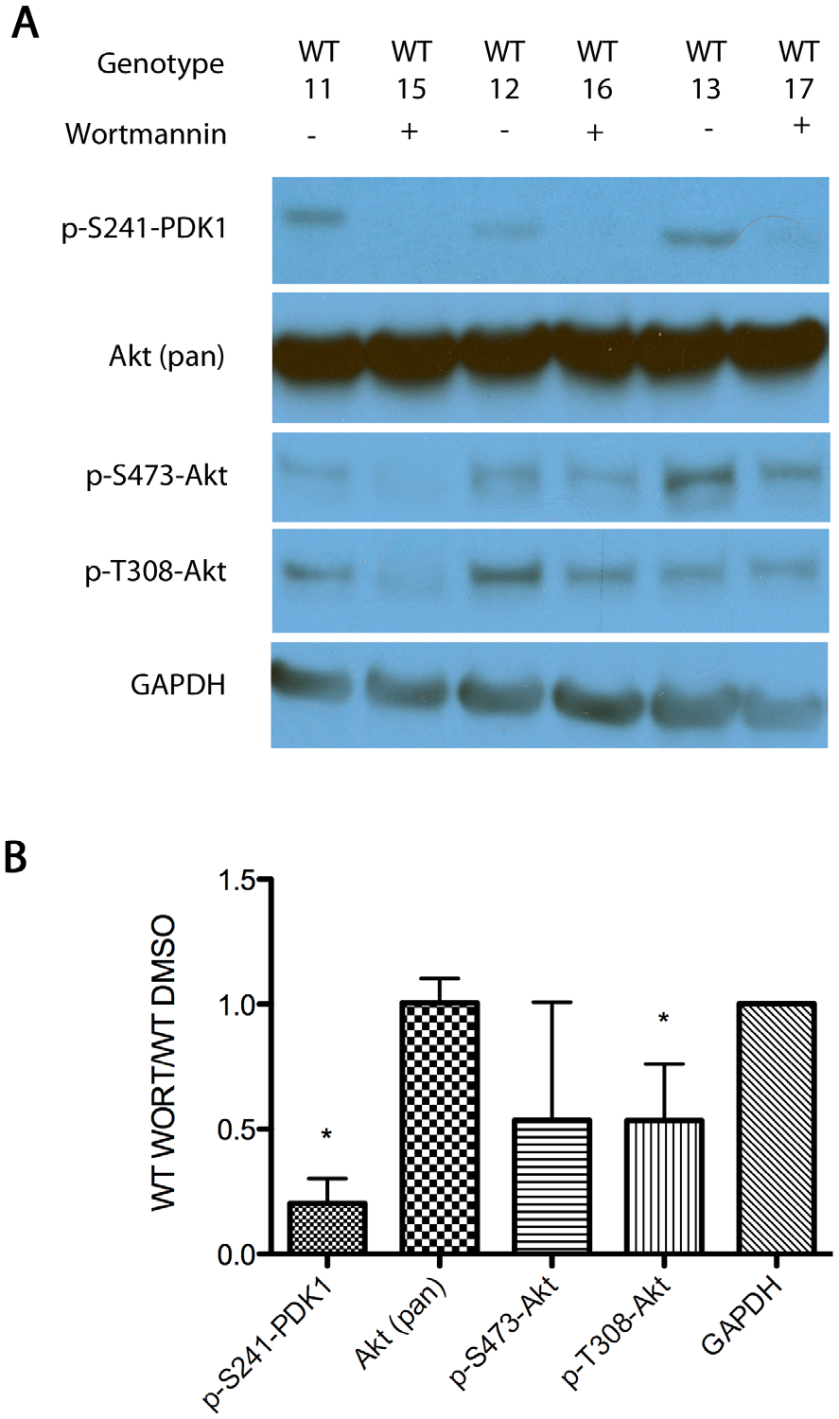
Western blot analysis of the phosphoproteins of the insulin-signaling cascade in homogenized whole-brains from male wild-type (WT) animals treated with wortmannin (+) or the DMSO control (-). Panel A: Representative Western blots from three DMSO control- and three wortmannin-treated animals. The molecular weight of each of the bands as determined in comparison with rainbow markers was consistent with the known weight of the target protein (Table 1). Each row represents an independent exposure. Panel B gives the results of densitometry from the entire group of 12 control and 17 wortmannin-treated animals. All bands were standardized to GAPDH controls to ensure equal protein loading. The error bars denote standard deviations; asterisks indicate statistical significance (p<0.05) by Student t-test between the wortmannin and DMSO control samples.

We then established the baseline effect of wortmannin treatment on 15 hour-fasted wild-type animals (Figure 6). Western blots showed that wortmannin significantly reduced PDK1 phosphorylation at Ser-241, an auto-phosphorylation site necessary for PDK1 activation and downstream signaling [32]. Although wortmannin reduced phosphorylation of Akt Thr-308 in these wild-type animals, it did not appear to statistically alter phosphorylation of the Ser-473 site. Additionally no effect was seen on mTOR phosphorylation (data not shown). These results show that orally administered wortmannin is an effective inhibitor of the insulin-signaling pathway in the brain as shown by the reduction in PDK1 and Akt phosphorylation [32], [33].

**Figure 7:**
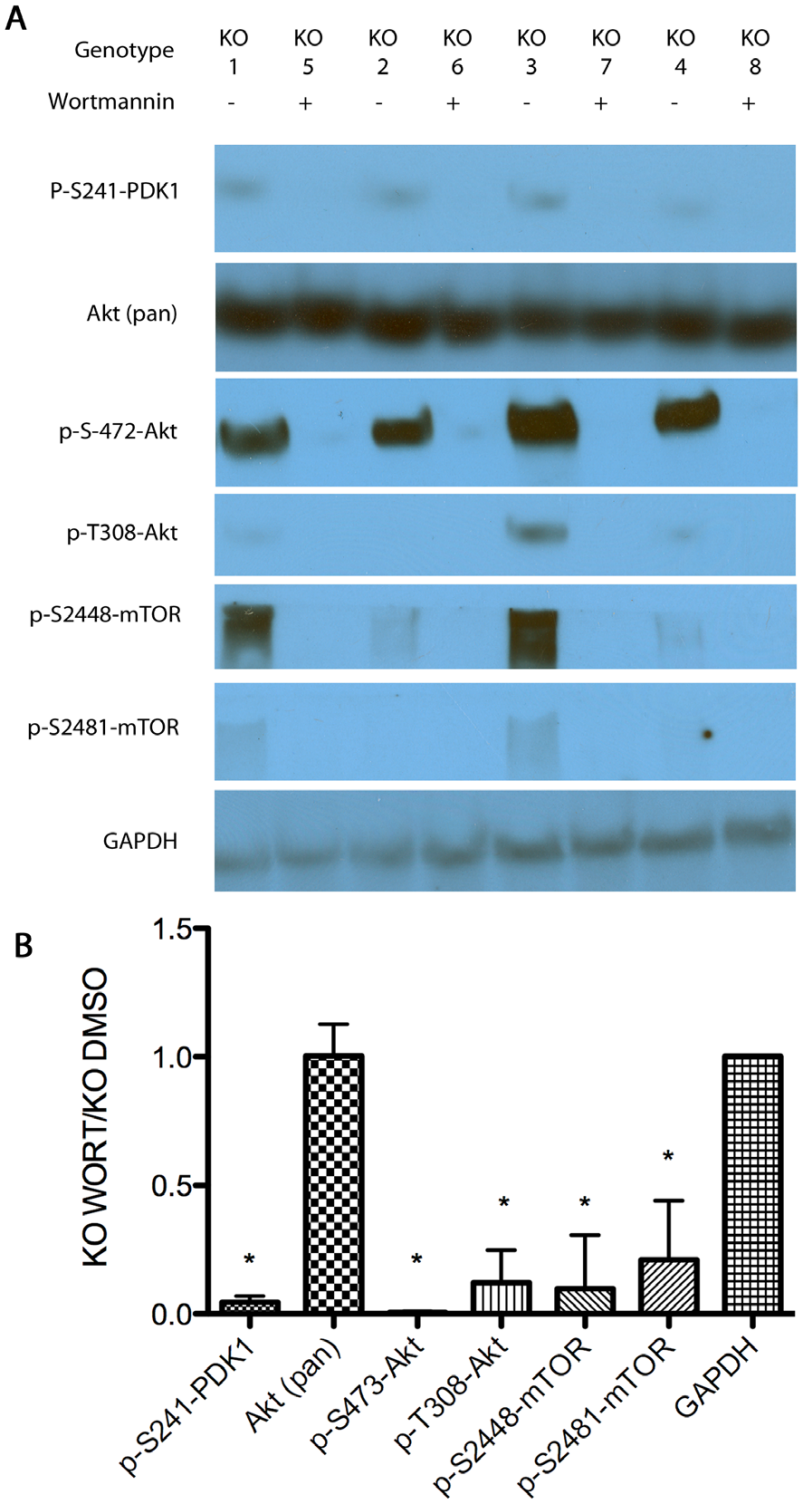
Western blot analysis of the insulin-signaling cascade in homogenized whole-brains from male animals. Panel A shows representative Western blots from four DMSO-control and four wortmannin-treated male *Pcmt1^−/−^* animals. The polypeptide molecular weight of each of the bands as determined in comparison with rainbow markers was consistent with the known weight of the target protein (Table 1). Each row represents an independent exposure. Panel B gives the results of densitometry from the entire group of 7 control and 11 wortmannin-treated animals. All bands were standardized to GAPDH controls to ensure equal protein loading. The error bars denote standard deviations; asterisks indicate a statistical significance (p<0.05) by Student t-test between the wortmannin and DMSO control samples.

Finally, we examined the effect wortmannin treatment had on *Pcmt1^−/−^* animals using Western blots with these brain extracts run side by side with *Pcmt1^−/−^* control extracts (Figure 7). In *Pcmt1^−/−^* mice, wortmannin decreased all of the phosphorylation sites related to canonical insulin signaling that were examined. Ser-241 of PDK1 had a nearly 10-fold decrease in phosphorylation. Downstream, phosphorylation of Thr-308 on Akt (the target of PDK1) was significantly reduced under wortmannin treatment. The Ser-473 site of Akt, phosphorylated by mTORC2, was also observed to be significantly decreased in wortmannin-treated *Pcmt1^−/−^* animals, suggesting insulin signaling mediated by Akt in the brains of *Pcmt1^−/−^* animals has been significantly ameliorated, a result also reflected in the reduced brain size of these animals compared to their *Pcmt1^−/−^* control counterparts. Auto-phosphorylation of mTOR as a result of PI3K/Akt signaling on the Ser-2481 site [34]–[35] as well as phosphorylation of Ser-2448 by the ribosomal protein S6 kinase [36], [37] is significantly decreased in *Pcmt1^−/−^* animals in the presence of wortmannin. As the mTOR phosphorylation sites were not seen to have decreased in wild-type animals subject to wortmannin treatment yet presented significant decreases in *Pcmt1^−/−^* animals this could potentially represent the point of convergence between the insulin signaling pathway and the isoaspartyl repair methyltransferase.

These data suggest *Pcmt1^−/−^* animals react to a reduction in insulin signaling in a distinctly different manner than wild-type mice. Wortmannin treated *Pcmt1^−/−^* mice show similar inhibition of PDK1 to wild-type animals, and yet show a much greater inhibition of mTOR and Akt1. This suggests that these sites are aberrantly activated in *Pcmt1^−/−^* mice, yet subject to the effects of wortmannin. Interestingly, despite larger reduction in insulin signaling upon wortmannin treatment in *Pcmt1^−/−^* mice, these animals still have larger brains than control treated wild-type animals. This observation suggests that the isoaspartyl methyltransferase could affect the insulin-signaling pathway downstream of Akt, or the existence of an alternative growth pathway that is activated in *Pcmt1^−/−^* animals.

### Effect of Wortmannin on the Accumulation of L-isoaspartyl Residues in Wild-type and Knockout *Pcmt1^−/−^* Mice

**Figure 8:**
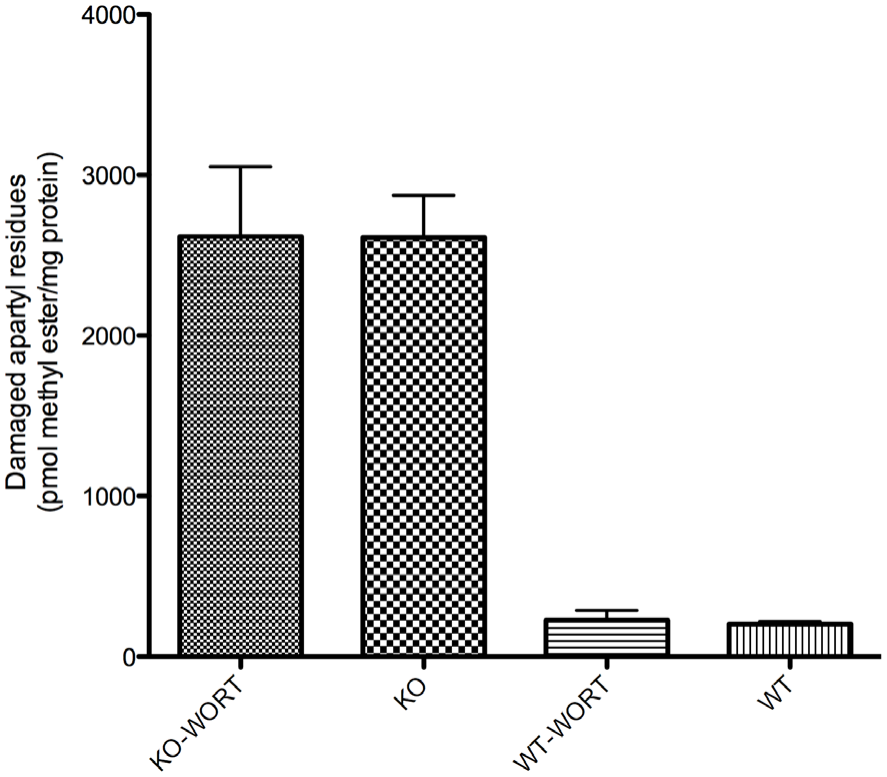
Quantitation of damaged aspartyl and asparaginyl residues in brain extracts. L-isoaspartyl residues arising *in vivo* in soluble brain polypeptides and proteins were labeled *in vitro* using recombinant human Pcmt1 and S-adenosyl- [^14^C] methionine. The resulting [^14^C] methyl esters were converted to [^14^C] methanol with sodium hydroxide and allowed to diffuse from filter paper into scintillation fluid, which was counted in a scintillation counter. There are significantly more damaged residues in KO brains than in WT brains, but no significant change due to wortmannin treatment (n = 7 for each experimental group).

Another phenotype that has been observed in Pcmt1−/− mice is the 8- to 14-fold accumulation of isoaspartyl residues in intracellular brain proteins [9], [10], [19]. Partial extension of the short lifespan of these mice was achieved by inserting a *Pcmt1* transgene on a weak neuron-specific promoter, and this was correlated with a partial decrease in isoaspartate accumulation in the brain [19]. To determine whether wortmannin’s protective effect is linked to isoaspartyl accumulation either through repair or by an increase in proteolytic degradation, we quantified the number of isoaspartyl residues in both *Pcmt1^−/−^* and wild-type animals. As expected, control *Pcmt1^−/−^* animals accumulated about 2500 pmol of methylatable isoaspartyl residues per milligram of protein while control wild-type animals had only approximately 200 pmol/mg (Figure 8). Interestingly, wortmannin had no effect on isoaspartyl accumulation in either wild-type or *Pcmt1^−/−^* animals, suggesting that the overall number of isoaspartyl residues in the brain proteins is not contributing to the prolonged survival of *Pcmt1−/−* mice afforded by wortmannin.

## Discussion

In this study we found evidence that the PI3K inhibitor wortmannin can decrease insulin signaling in both *Pcmt1^−/−^* as well as wild-type mice, decrease the enlarged brain phenotype typical of *Pcmt1^−/−^* animals, and prolong the survival of *Pcmt1^−/−^* mice. Our observations suggest that the *Pcmt1^−/−^*activated growth pathways are confined to brain tissue as we find an approximate 20% increase in brain mass over wild-type animals at 45 days of age, yet a reduced overall body weight. This growth paradox highlights the importance of PCMT1 in the brain and suggests a role for this enzyme in brain growth and development. It is currently unknown, however, whether unrepaired isoaspartyl residues are acting as molecular switches triggering brain growth or whether the methyltransferase itself has a moonlighting role in mammalian development and growth. Our observation that a near complete reduction of phosphorylation of PDK1, mTOR and Akt1 does not completely abolish the enlarged brains of *Pcmt1^−/−^* animals suggests that the convergence of this methyltransferase with the insulin signaling pathway either occurs at, or downstream of, the kinase Akt. Alternatively, PCMT1 could be influencing brain growth through a different, Akt independent, growth pathway. For example, Kosugi et al. have shown that PCMT1 activity is also required for normal signaling through the MAPK pathway in cultured human embryonic kidney cells upon addition of EGF [38]. Additionally, although wortmannin was able to partially decrease the size of the enlarged brains of *Pcmt1^−/−^* animals, it succeeded only in prolonging the time until death (seizure onset), not preventing the early death phenotype. This suggests that the enlarged brain phenotype of *Pcmt1^−/−^* mice may be a contributing factor toward, but not the entire underlying cause of, the seizure phenotype and early death these mice experience.

The Akt kinase is at the center of the insulin-signaling pathway [30]. Interestingly mice have three genes expressing highly similar forms of the enzyme designated Akt1, Akt2, and Akt3 [30]. Akt1 is expressed ubiquitously outside of the brain and is responsible for global growth [39]. Akt2 is primarily responsible for maintaining insulin sensitivity to changing blood glucose levels and is confined to brown fat, skeletal muscle and the β-islet cells of the pancreas [40]. Akt3, of most interest to the present study, is expressed only in neurons and testis, and when genetically deleted has been shown to decrease brain size, indicating that it is largely responsible for brain growth and development [41]–[42]. Conversely, mutations leading to constitutive activation of this gene result in an enlarged brain and seizure phenotype [43], not dissimilar from the phenotypes observed in our *Pcmt1^−/−^* mice [9], [10], [11], [12], [14], [19]. Our findings suggest that the Akt3 enzyme presents a brain-specific convergence point between PCMT1 and growth pathways and could provide a unique age-sensitive point of regulation of Akt3, either by an isoaspartyl “switch” or through interaction with PCMT1 itself.

**Figure 9:**
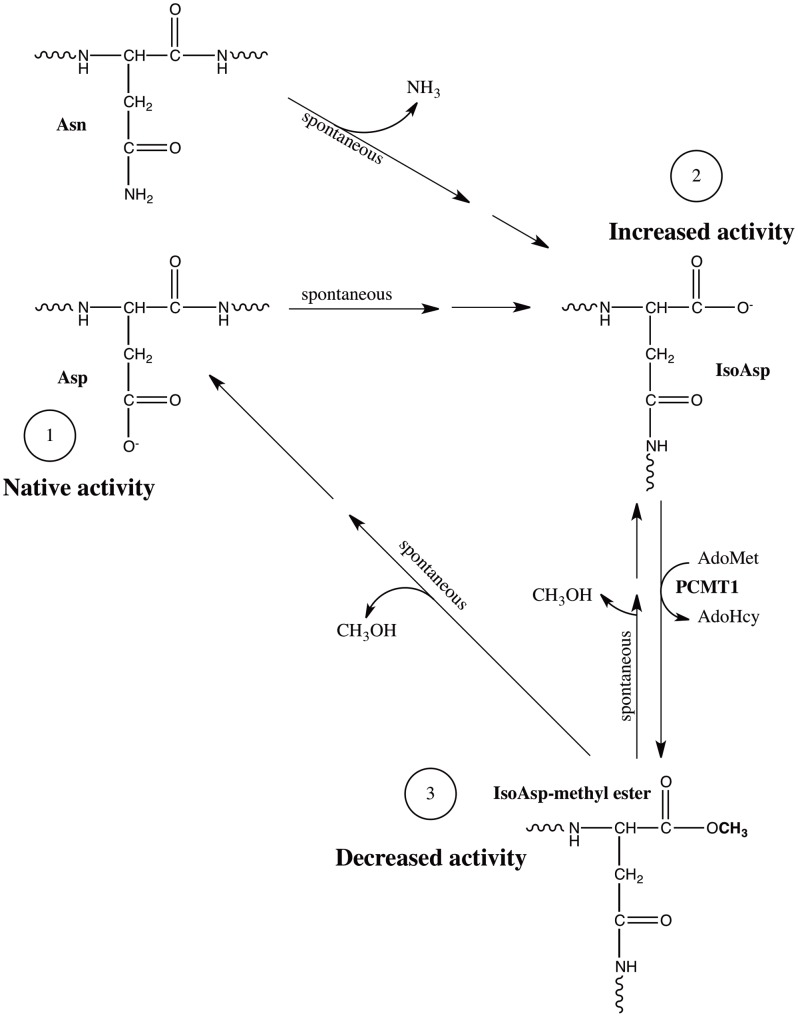
Simplified illustration of a hypothetical isoaspartyl switch. (1) The original Asp and Asn residues set a native activation baseline in a given protein. (2) Spontaneous deamidation or isomerization of Asn and Asp residues**,** respectively, through succinimide intermediates (not shown) yield isoaspartyl residues and potentially more active enzymes or better substrates for activating kinases. (3) AdoMet-dependent methylation of the isoaspartyl residue by PCMT1 yields an isoaspartyl-methyl ester, a potentially less active form. Spontaneous de-esterification via succinimide intermediates (not shown) can restore the active isoaspartyl-containing form (2), or result in reversed isomerization, returning the residue to the native aspartyl configuration (1), restoring native activity, or altered activity if the residue was initially asparagine. Such a switch may control the phosphorylation or activation of Akt3 or other proteins in the signaling pathways.

The possibility that Akt3 contains an isoaspartyl-regulated switch like that proposed for BCL-xL [44], [45] and p53 [46] is supported by the fact that Akt3 has 9 additional potential isoaspartyl-forming asparagine and aspartic acid residues compared to Akt1, and 7 potential isoaspartyl-forming residues more than Akt2. Interestingly, some of these residues in Akt3 flank the crucial hydrophobic motif that is necessary for mTOR binding and activation [47], [48], [49]. The Akt3 isoform has also been linked to aberrant brain growth and seizure onset in humans [50]. We can propose the possibility that the isoaspartyl forms of Akt3 are more active than the methylated or non-isomerized forms (Figure 9); this model would account for the activation of the insulin-signaling pathway in *Pcmt1^−/−^* animals. Although other growth pathways have been shown to be sensitive to, or regulated by, PCMT1 activity such as the MAPK/ERK pathway [51] [38] [52]–[54], they do not rely on brain specific constituents and provide unlikely explanations for the aberrant brain growth phenotype observed in *Pcmt1^−/−^* mice.

**Figure 10:**
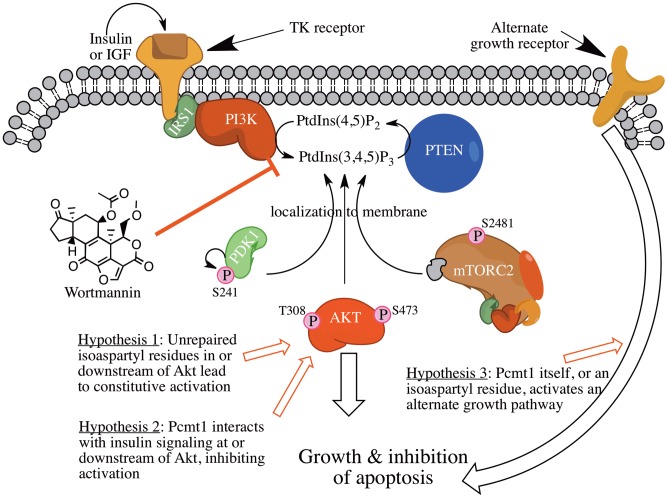
Possible points of interaction between *Pcmt1* and the upstream insulin-signaling pathway. Wortmannin inhibits PI3K class kinases and has been shown to inhibit insulin signaling. The elevated insulin signaling in *Pcmt1^−/−^* animals could arise from aberrant Akt activation (hypothesis 1 or 2), and/or aberrant interaction between Akt1 and mTOR (hypothesis 1 or 2) or activation of an alternate growth pathway (hypothesis 3).

The hypothesis of a brain specific isoaspartyl molecular switch regulating mTOR/Akt activation (Figure 10) correlates with our quantitative analysis of the increased phosphorylation and activation of mTOR and Akt in DMSO-treated *Pcmt1^−/−^* mice as compared to DMSO-treated wild-type animals and is currently under investigation. Additionally it appears that phosphorylation of the mTOR dependent Ser-473 site of Akt as well as mTOR itself exhibited a different reaction to the drug wortmannin in *Pcmt1^−/−^* mice with dramatic decreases in phosphorylation, a change not seen in wild-type animals. Despite wortmannin reducing phosphorylation of these enzymes, *Pcmt1^−/−^* mice on wortmannin still exhibit enlarged brains, suggesting the possibility that this pathway remains somewhat activated, potentially due to the unique isomerization or deamidation prone-residues neighboring the hydrophobic motif of Akt3.

This model suggests a unique post-translational control point governing brain Akt/mTOR interaction that could theoretically be responsible for elevating the growth pathways in *Pcmt1^−/−^* mice. Additionally this model implicates PCMT1 and isoaspartyl residues as age-related switches regulating entry into apoptotic pathways as has recently been shown in BCL-xL [45] [44] and p53 [46]. Additionally, wortmannin itself has been shown to trigger apoptosis through inhibition of PI3K class kinases [55]–[56] in a manner somewhat opposite of Pcmt1. These data present a striking opportunity for further research into the role of these pathways and apoptosis in seizure onset within this mouse model.

Our finding that *Pcmt1^−/−^* mice are smaller than wild-type animals at the time of weaning, but gain weight at a similar rate post-weaning suggests at least two possibilities. First, *Pcmt1^−/−^* mice could have a defect in early development limiting their size but still have normal post-weaning development. Second, they could suffer neurological deficits limiting their milking instinct, leading to competition from wild-type littermates for breastfeeding time, and thus decreasing developing body mass due to nutrient shortage. This hypothesis would support the observation of normal development post-weaning, as *Pcmt1^−/−^* animals would not face littermate competition for the easily accessible chow diet. A mouse line in which *Pcmt1* could be knocked out at 21 days of age using a CRE-Lox system would help distinguish between the roles of PCMT1 in developing versus weaned animals.

## Supporting Information

Figure S1 Effect of wortmannin (WORT) on the post-weaning weight gain of wild-type (WT) and *Pcmt1^−/−^* (KO) mice. This figure shows the averaged absolute weights of the same animals whose relative weight gain is illustrated in Figure 2. In panels A-D, wild-type weight gains are shown in closed squares and *Pcmt1^−/−^* weight gains are shown in open circles. In panels E-H, weight gains of wortmannin-treated animals are shown in closed squares while those of DMSO-treated control mice are shown in open circles.
(TIFF)

Figure S2 Comparison of survival of untreated male (n = 51) and female (n = 57) Pcmt1*^−/−^* (KO) mice from day 21 of weaning. Untreated animals that died prior to 50 days of age were plotted on a Kaplan-Meier curve. No significant difference was observed between sexes. P = 0.334 by the Gehan-Breslow-Wilcoxon test.
(TIFF)
