# Supplementary figures and images for: Wortmannin Reduces Insulin Signaling and Death in Seizure-Prone Pcmt1−/− Mice
Source: PLoS One. 2012 Oct 5;7(10):e46719. doi: 10.1371/journal.pone.0046719 (PMC3465263; doi:10.1371/journal.pone.0046719)

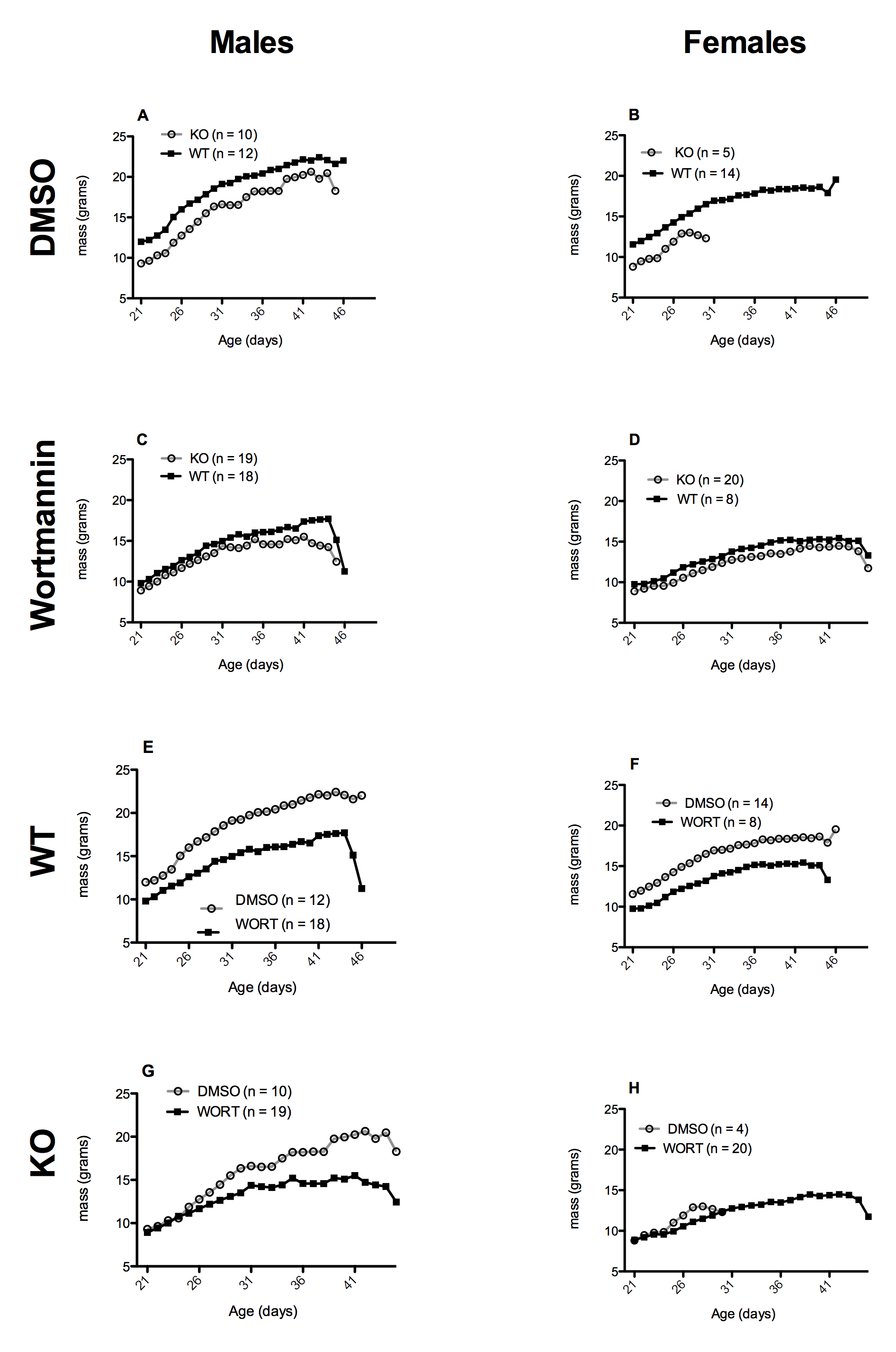

Supplement: Figure S1 — Effect of wortmannin (WORT) on the post-weaning weight gain of wild-type (WT) and Pcmt1−/− (KO) mice. This figure shows the averaged absolute weights of the same animals whose relative weight gain is illustrated in Figure 2. In panels A-D, wild-type weight gains are shown in closed squares and Pcmt1−/− weight gains are shown in open circles. In panels E-H, weight gains of wortmannin-treated animals are shown in closed squares while those of DMSO-treated control mice are shown in open circles. (TIFF) [file pone.0046719.s001.tiff]

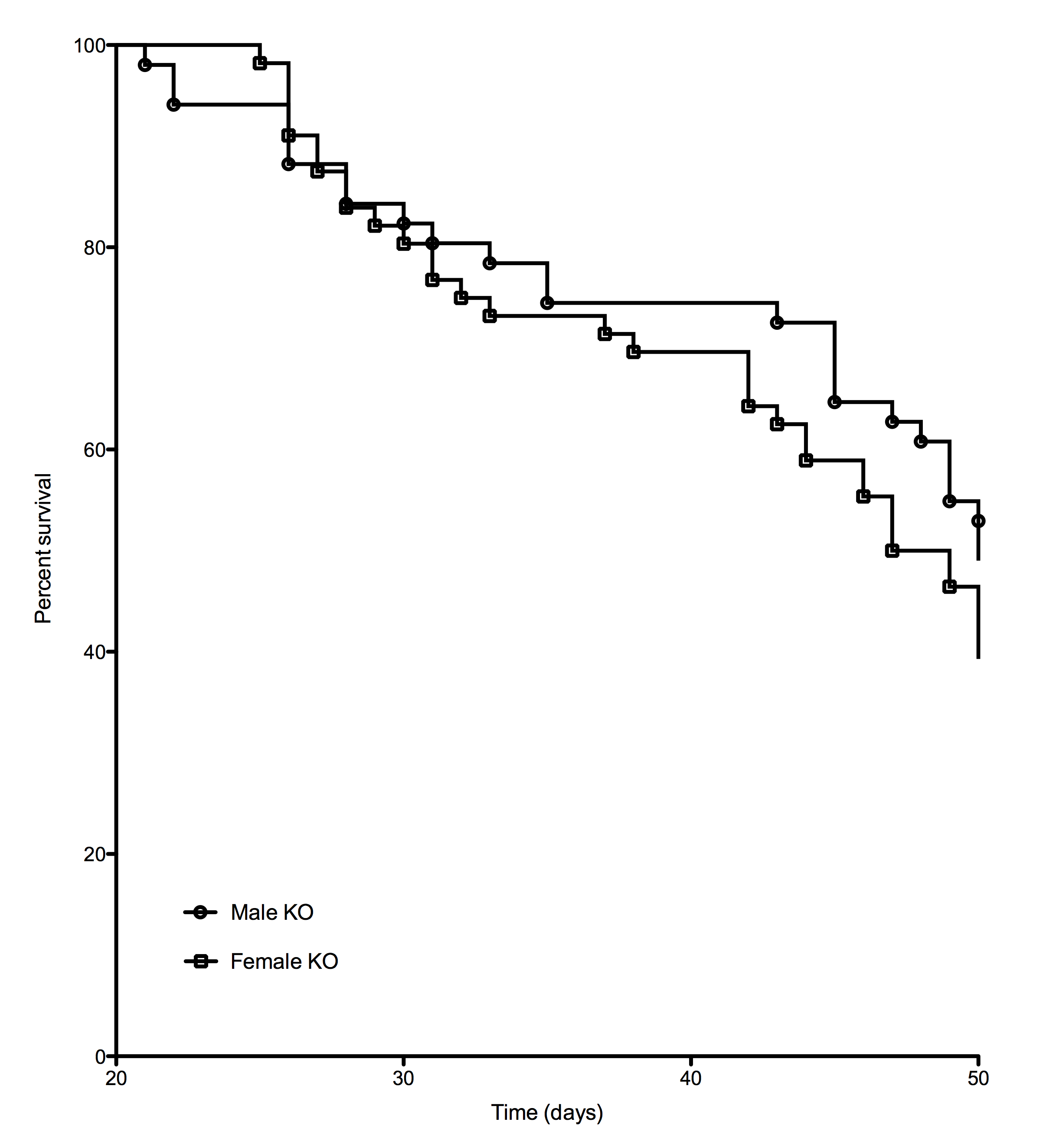

Supplement: Figure S2 — Comparison of survival of untreated male (n = 51) and female (n = 57) Pcmt1−/− (KO) mice from day 21 of weaning. Untreated animals that died prior to 50 days of age were plotted on a Kaplan-Meier curve. No significant difference was observed between sexes. P = 0.334 by the Gehan-Breslow-Wilcoxon test. (TIFF) [file pone.0046719.s002.tiff]
